# The Safety Climate and Patient Safety Activities in Mental Health Nurses: The Mediating Effect of Safety Control

**DOI:** 10.3390/healthcare12121181

**Published:** 2024-06-12

**Authors:** Jiyeong No, Kyoungsook Lee

**Affiliations:** 1 Department of Nursing, Jinju Health College, Jinju 52655, Republic of Korea; nsophiay@nate.com; 2 Department of Nursing, University of Ulsan, Ulsan 44610, Republic of Korea

**Keywords:** psychiatry, nurse, safety climate, patient safety

## Abstract

This study aimed to investigate the mediating effect of safety control on the relationship between safety climate and patient safety management activities for mental health nurses. A survey was conducted on 177 nurses working at mental hospitals in G-do from 28 July 2023 to 15 August 2023. Data were analyzed using the SPSS/WIN 27.0 and Sobel test. Significant relationships were found between safety climate and safety control (r = 0.40, *p* < 0.001), safety climate and patient safety management activities(r = 0.40, *p* < 0.001), and safety control and patient safety management activities (r = 0.43, *p* < 0.001). Additionally, safety control partially mediated the safety climate and the patient safety management activities (Z = 3.63, *p* < 0.001). Therefore, programs that increase safety control and create a safety climate need to be developed to promote patient safety activities of mental health nurses.

## 1. Introduction

Ensuring patient safety is paramount in the dynamic and demanding healthcare field. Mental health nurses play a crucial role in providing care for individuals facing complex psychological challenges [1]. Since the introduction of the Hospital Accreditation System in 2013, mental hospitals have established and managed patient safety guidelines to prevent patient safety accidents [1], and the quality management of medical service has been emphasized. Generally, patients with psychiatric problems who need hospitalization are treated in closed wards. Those with severe psychiatric problems tend to be hospitalized for a long time, thereby being at risk of safety accidents for longer [2]. Unexpected accidents, such as the patient’s aggressive behavior and self-injuries, and safety accidents, including suffocation, falls, and obsessive compulsion, frequently occur in mental hospitals due to inpatient characteristics [3]. Additionally, patients have a high risk of falls because of various problems caused by mental illnesses, such as cognitive impairment, mania-induced hyperactivity, depression, deliration, and vertigo, after psychoactive drug administration. Therefore, patient care and safety accident prevention should be focused on [4]. Mental wards are different from general wards. Patients in mental wards highly depend on medical staff since they have a closed environment. Additionally, an effort to secure environmental facilities in terms of safety management is necessary because of the environmental characteristics of the wards [5]. Various factors influenced the effectiveness of patient safety activities within mental health settings, with safety control and safety climate emerging as key determinants.

Patient safety accidents are closely related to nursing jobs, and nurses are the last healthcare providers to filter errors [6]. Therefore, an effort to guarantee patient safety is necessary. Patient safety activities are actions and activities taken by medical service organizations and staff to prevent possible disasters or accidents of patients, aiming to reduce, remove, and prevent risk factors [7]. Clinical risk management for falls in healthcare environments involves identifying, assessing, and mitigating the risks associated with falls to improve patient safety and reduce the incidence and severity of fall-related injuries [8]. In addition, factors influencing the patient safety activities of nurses working at mental hospitals need to be considered to determine ways to improve them.

Safety climate refers to the perception of employees regarding the importance of safety within their organization [9]. In mental health nursing, it encompasses the attitudes, beliefs, and values related to patient safety among nurses and other healthcare professionals. A positive safety climate indicates a strong emphasis on safety, open communication about safety concerns, and a supportive environment for reporting errors or near misses [10].

Moreover, safety control is a personal factor affecting patient safety. It is the cognitive ability to draw secure outcomes while executing a job [11]. In addition, it is not made reflectively by the process of individuals controlling their behavior but by a sense of self-control that detects and evaluates their behavior [12]. Self-control helps in decision-making by finding multiple ways to generate a secure state and considering safety as the top priority when factors impeding safety exist. The addition of safety consciousness and the concept of safety to self-control generates safety control [13]. Furthermore, safety control contributes to the fulfillment of safety behavior in jobs that highly require finishing a task and entail large responsibilities, such as the nursing job [14]. The higher the safety control, the lower the negative safety index [15], and the more positive its influence on personal safety and patient safety activities [16].

Generating a positive safety climate, where members of mental hospitals can provide appropriate responses and stable treatment in caring for patients, is necessary regarding patient safety activities [17]. Enhancing an effort for safety is possible when an organization and its members share a positive perception of patient safety management [18]. The rate of safety accidents reduces when the safety climate is positive [9], patient safety activities are less performed otherwise. This is because the fear of personal blame and punishment results in poor reports and follow-up measures [19]. Understanding the dynamics at work in mental healthcare settings is crucial, not only for the welfare of patients but also for the well-being of the nursing workforce. The mental health nursing profession is characterized by its demanding nature, frequently accompanied by high levels of stress and emotional labor. Hence, identifying factors that can enhance the safety culture and support patient safety activities among mental health nurses has far-reaching implications for both patient care and the job satisfaction and retention of these dedicated healthcare professionals.

Regarding the previous studies on patient safety activities of nurses, studies were conducted on related variables, such as the perception of patient safety culture [20] and safety competency [21]. Furthermore, studies were conducted on subjects, such as nurses at general hospitals [17], nurses at mid and small-sized hospitals [22], and nurses in operating rooms [23]. The study on mental health nurses analyzed the relationship between patient safety activities and the perception of patient safety culture [2]. Nurses’ patient safety activities are important for patient safety, and safety climate and safety control are related to such activities. However, most of the studies focused on factors influencing patient safety activities, with no research confirming the mediating effect of safety control. The current study aimed to contribute to the broader discourse on healthcare safety and inform strategies for improving patient outcomes in the complex and critical field of mental healthcare by exploring the association between safety control and safety climate. This study also sought to verify the mediating effects of safety control in the impact of safety climate on the patient safety nursing activities of mental health nurses.

These research findings formed the basis to hypothesize that (a) the general characteristics of mental health nurses will affect patient safety activities; (b) there will be associations among safety control, safety climate, and patient safety activities; (c) safety climate and safety control will affect patient safety activities; and (d) in the relationship between safety climate and patient safety activities, safety control will have a mediating effect.

## 2. Methods

### 2.1. Study Design

**Figure 1:**
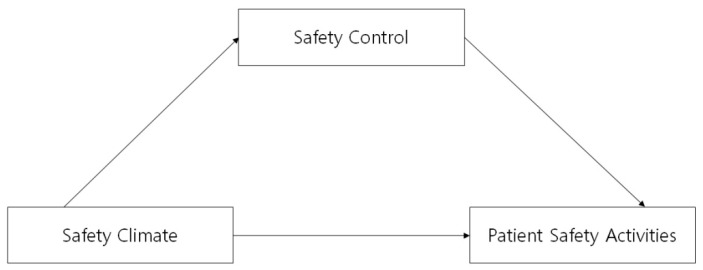
Conceptual framework.

This study is a descriptive survey research to verify the degree of safety climate, safety control, and patient safety activities of mental health nurses, correlations among variables, and determine the factors influencing patient safety activities (Figure 1).

### 2.2. Study Participants

This study included nurses who are working in nine mental hospitals in G-do and agreed to participate in this study voluntarily. The G-power 3.1.9.4 program was used to estimate the number of study participants. Regression analysis with a significance level of 0.05, explanatory power of 0.95, medium effect size of 0.15, and the number of independent variables of 10 revealed the minimum number of 172 samples. A total of 192 copies of a structured questionnaire were distributed, considering the dropout rate (10%), and 182 copies were collected. Five copies with insincere answers were excluded. Finally, 177 copies were analyzed.

### 2.3. Study Population

The institutional review board (IRB) of U-university approved this study before being conducted (No. 1040968-A-2023-015). The study participants who heard the purposes of this study and agreed on study participation were requested to answer the questions in the self-report questionnaire. Data have been collected from 28 July to 15 August 2023. Before data collection, researchers called the director of the nursing department in each mental hospital and received permission for the questionnaire. The explanation of study purposes, anonymity, and confidentiality were given to the study participants, considering the ethical aspects of the study participants. The study participants were allowed to give up their participation anytime unless they wanted and were informed that the collected data were not going to be used for other purposes than the original ones. After the study participants signed the written consent, the questionnaire survey was conducted. Additionally, the participants were notified of the plan that the questionnaire data would be accessed by this researcher only and be destroyed fully after the end of the study. The questionnaire was completed for approximately 15 min. A small return was provided for the participants thereafter. The researchers organized and analyzed the data while maintaining participant anonymity. The consent form and survey data will be kept for 3 years, and the coded data will be retained for at least 5 years before being deleted.

### 2.4. Measures

#### 2.4.1. Safety Climate

The Patient Safety Climate in Healthcare Organization developed by Singer et al. [24] and translated by Hwang [25] was used for safety climate. It has 33 items on the 5-point Likert scale which was based on the 5-point Likert scale from 1 point to 5 points (1: strongly disagree, 2: disagree, 3: neutral, 4: agree, and 5: strongly agree). The higher the score, the more safety climate. During the development, the reliability of the scale was Cronbach’s α of 0.44–0.80. The study by Hwang [25] revealed Cronbach’s α of 0.91 while the present study reported Cronbach’s α of 0.92.

#### 2.4.2. Safety Control

The measurement scale developed by Anderson et al. [11] and translated by Chung [13] was used for safety control. It has seven items on the 5-point Likert scale which was based on the 5-point Likert scale from 1 point to 5 points (1: strongly disagree, 2: disagree, 3: neutral, 4: agree, and 5: strongly agree). The higher the score, the more safety control. The study by Anderson et al. [11] revealed the reliability of the scale as Cronbach’s α of 0.85; the study by Chung [13] presented Cronbach’s α of 0.84; the current study reported Cronbach’s α of 0.81.

#### 2.4.3. Patient Safety Activity

The scale developed by Lee [26] and modified by Kim and Lee [2] in line with the settings of mental hospitals was used for patient safety activities. The subcategories of the scale included patient identification, prescription and medication, environmental safety, infection/fall/pressure ulcer prevention, coping emergency, and coping psychiatric problems situation, consisting of 32 items. Each item is based on the 5-point Likert scale which was based on the 5-point Likert scale from 1 point to 5 points (1: strongly disagree, 2: disagree, 3: neutral, 4: agree, and 5: strongly agree). The higher the score, the more patient safety activities. The study by Lee [26] revealed the reliability of the scale as Cronbach’s α of 0.95; the study by Kim and Lee [2] presented Cronbach’s α of 0.95; the present study reported Cronbach’s α of 0.91.

### 2.5. Data Analysis

The collected data were analyzed with Statistical Package for the Social Sciences v.27.0. Details about the analysis method are as follows. First, the participants’ safety control, professional autonomy, safety climate, and patient safety activities were analyzed with the mean and standard deviation. Second, the differences in the participants’ patient safety activities were analyzed with a *t*-test and analysis of variance, depending on their general characteristics. The Scheffé test was conducted as a post-hoc test. Third, the correlations between participants’ safety climate, safety control, and patient safety activities were analyzed with Pearson’s correlation coefficient. Fourth, multiple regression analysis was performed to analyze the influential factors on the subjects’ patient safety activities. The multiple regression analysis-based three-step mediation analysis approach of Baron and Kenny [27] was used to analyze the mediating effect of safety control in the association between the subjects‘ safety climate and patient safety activities. The Sobel test was conducted to determine the statistical significance of the mediating effect. To ensure the statistical analysis is accurate and reliable, a professional statistician evaluated and approved the tests. In addition, in relation to statistics, we were consulted by Dr. Kim, who has taught nursing statistics at the nursing department of U University in G City for more than 10 years.

## 3. Results

### 3.1. Patient Safety Activities Based on General Characteristics

Of the 177 mental health nurses, 156 (88.1%) participants were women. Their average age was 46.55 ± 7.39 years and 65 (36.7%) nurses were aged 31–40 years. Overall, 104 (58.8%) participants were married and 92 (52.0%) participants graduated from college. Regarding psychiatric ward experience, 50 people (28.2%) were the most experienced between three years and less than six years, but groups with less than three years, more than six years, and less than 10 years were similar. In terms of subjective health status, 90 (50.8%) participants replied “moderate or poor”, 99 (55.9%) participants had experienced violence within one month and 95participants (53.7%) replied “satisfied” about job satisfaction.

Table 1 shows the analysis of the participants’ patient safety activities depending on their general characteristics. The patient safety activities were not significantly different depending on the general characteristics.

### 3.2. Degrees of Nurses’ Safety Climate, Safety Control, and Patient Safety Activities

Table 2 presents the values of the participants’ safety climate, safety control, and patient safety activities. The mean ± standard deviation (SD) of safety climate scored 3.48 ± 0.54 of 5 points in mental health nurses. The mean ± SD of safety control scored 3.65 ± 0.80 of 5 points. The mean ± SD of the safety climate of the hospital contribution scored 3.37 ± 0.63 points and the work-unit contribution scored 3.54 ± 0.57 points in the subcategories. The mean ± SD of patient safety activities scored 3.87 ± 0.47 of 5 points. Patient identification prescription and medication environment safety, infection/fall/pressure ulcer prevention, coping emergency, and coping psychiatric problems and situations scored 3.83 ± 0.72, 3.87 ± 0.61, 3.73 ± 0.83, 3.88 ± 0.60, 3.90 ± 0.64, and 3.95 ± 0.73 points in the subcategories, respectively.

### 3.3. Correlations between Variables

The correlations between the participants’ safety climate, safety control, and patient safety activities were analyzed (Table 3). Patient safety activities had statistical and significant positive correlations with safety climate (r = 0.40, *p* < 0.001) and safety control (r = 0.43, *p* < 0.001). Safety control had a positive correlation with safety climate (r = 0.40, *p* < 0.001).

### 3.4. Mediating Effect of Safety Control on the Relationship between the Study Participants’ Safety Climate and Patient Safety Activities

Regression analysis was performed on the mediating effects (Table 4). Prior to verifying the mediating effect, the basic assumptions of regression analysis were reviewed, and there was no autocorrelation because the Durbin–Watson value was 2.02, which was closer to 2, and the tolerance limit was 0.59 to 0.98, which was more than 0.1, and the variance inflation factor was between 1.02 and 1.69, which was less than 10, so there was no problem of multicollinearity among predictors, which satisfied all the assumptions of regression analysis.

**Figure 2:**
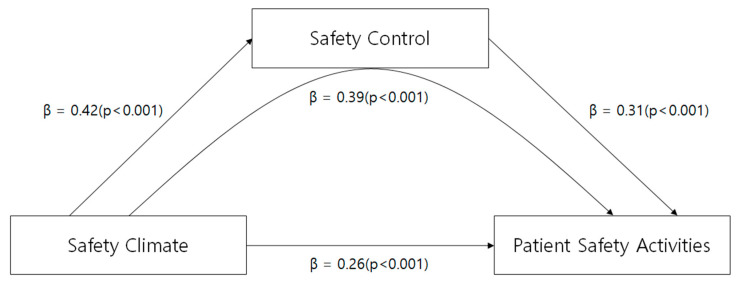
The mediating effect of safety control.

The mediating effect of safety control on the association between safety climate and patient safety activities on mental health nurses was analyzed. The independent variable “safety climate” statistically and significantly affected the mediating variable “safety control” in step 1 (β = 0.42, *p* < 0.001). The independent variable “safety climate” statistically and significantly influenced the dependent variable “patient safety activities” in step 2 (β = 0.39, *p* < 0.001). Safety climate and the mediating variable “safety control” as independent variables, and “patient safety activities” as a dependent variable were used in step 3. Thus, safety climate and safety control significantly influenced patient safety activities (β = 0.26, *p* < 0.001). A comparison of regression coefficients demonstrated that the influential power of the independent variable “safety climate” on patient safety activities in step 2 (β = 0.39) was lower than that in step 3 (β = 0.26). Therefore, safety control partially mediated the association between safety climate and patient safety activities. The R^2^-valuethat represents explanatory power was 18%, 15.2%, and 22.9% in steps 1, 2, and 3, respectively. The Sobel test was performed to verify the significance of the mediating effect coefficients. The result revealed that the absolute value of Z was larger than 1.96; thus, the mediating effect of safety control was statistically significant (Z = 3.66, *p* < 0.001) (Figure 2).

## 4. Discussion

Patient safety is a crucial determinant of healthcare quality [28]. This study was conducted to determine mental health nurses’ safety control, safety climate, and patient safety activities and revealed the influential factors on patient safety activities.

This study revealed that safety control scored 3.48 out of 5 points. The study by Kim who used the same scale revealed that the safety control of nurses working at small and mid-sized hospitals scored 3.74. Thus, the safety control of the participants in this study scored lower. The reason why mental health nurses’ safety control scored lower was that, unlike general hospitals, mental hospitals face special safety accidents caused by unexpected behaviors, such as safety problems, suicide, violence, and escape, of patients with psychiatric problems. The nurses caring for patients should sensitively practice safety execution, and a level of their personal work control should guarantee patient safety [29]. Patients admitted to mental hospitals have a high risk of safety accidents due to the lowered cognition induced by mental illnesses and the side effects of psychoactive drugs. Therefore, plans and programs to improve mental health nurses’ safety control are necessary to be developed.

Safety climate scored 3.48 out of 5 points. It scored 3.14 in the same scale-based study on nurses in the operating room. The study on hospital nurses by Kim and Kang [17] revealed a score of 3.58. Hence, the score of safety climate was different. This is because of the different characteristics of each hospital or department depending on patient illnesses. Providing sufficient resources, training for safety, and establishing a safety climate to communicate and cope with risk factors or mistakes is necessary to prevent patient safety accidents or make improvements. The attitudes of the management and direct supervisors are important regarding safety climate [17]. Therefore, educating them in terms of safety climate is necessary. The safety climate of the hospital scored 3.37 and that of the department scored 3.54 in the subcategories. This indicates that the safety climate of the hospital was a little more positive than that of the department. However, the study on nurses at mental hospitals by Jang and Shin [5] revealed that the safety climate of the hospital scored higher than that of the department, which was different from the result of this study.

Patient safety activities scored 3.87 out of 5 points. The study on nurses at psychiatric medical institutions by Kim and Lee [2] revealed a score of 3.9. Thus, the scores of patient safety activities were similar. In the subcategories, coping psychiatric problems and situations scored the highest at 3.95, and environment safety scored the lowest at 3.73. The study by Kim and Lee [2] drew the same result. Therefore, mental health nurses take many patient safety activities, such as the evaluation and prevention of patient suicide, violence, and leave without permission, considering the specialties of the psychiatric department, and restriction to carrying dangerous items. Focusing on the activities to build a safe environment is necessary because mental health nurses perceive less environmental safety. This study revealed no difference in patient safety activities depending on the general characteristics of the participants. However, the study by Jang and Shin [5] revealed that these activities were different depending on age and career in the psychiatric department. Considering various characteristics is necessary because of a few studies on mental health nurses’ patient safety activities.

Mental health nurses’ safety control and safety climate had significantly positive correlations with their patient safety activities. The study on nurses at long-term nursing facilities by Seo and Do [30] revealed that the higher the nurses’ safety control, the more they had patient safety activities. It was similar to the result of this study. As a safe manager, nurses play a role in protecting patients in the closest distance. Hence, the importance of safety control has been emphasized [22]. Nurses’ safety control helps to make clear judgments on the behaviors that damage patient safety, and how each one perceives the issue of patient safety affects patient safety activities [13]. The positive correlation between the sense of safety control, safety climate, and patient safety activities among mental health nurses highlights the critical role of organizational culture and support in ensuring patient safety. Healthcare organizations can enhance patient safety outcomes and empower their nursing staff to actively contribute to patient safety management by addressing these factors and fostering a culture of safety.

The study by Lee and Ryu [31] revealed that their patient safety activities were improved as the safety climate perceived by nurses was more positive. It was similar to the result of this study. Safety climate is affected by the culture of an organization [31]. The study by Jang and Shin [5] revealed that the more the nurses had patient safety activities, the more they perceived the safety climate at mental hospitals more positively. It was consistent with the result of this study. To improve mental health nurses’ patient safety activities, building an environment where it is possible to form opinions freely if patient safety accidents occur at mental hospitals, or if something negatively influences patient treatment is necessary. Additionally, solutions and appropriate follow-up measures, rather than reprimand and reproach, are necessary. Open communication and members’ efforts are considered helpful in building a positive safety climate.

The influential factors on patient safety activities were safety control and safety climate, and the total explanatory power was 30.7%. The study on nurses at long-term nursing facilities by Seo and Do [30] and the study on nurses at small and mid-sized hospitals by Kim [22] revealed that safety control was a significant factor influencing patient safety activities. Preparing a detailed plan to increase safety control as personal safety competency is necessary to promote patient safety activities. Generally, safety knowledge [32], personal competency, and education [33] are critical factors of safety control. Therefore, the program utilizing the factor is considered effective. Safety control measures, which encompass policies, procedures, and practices implemented within healthcare organizations to enhance patient safety, were found to have a substantial positive influence on the engagement of mental health nurses in patient safety activities. This result indicates that mental health nurses are more likely to actively participate in patient safety initiatives when robust safety control mechanisms are in place.

The safety climate within healthcare settings, particularly in mental health facilities, is crucial for ensuring the well-being of patients and healthcare professionals. This study explored the association between safety climate and patient safety activities among mental health nurses, with a focus on the mediating role of safety control. These findings provide valuable insights into the dynamics of safety climate and its impact on patient safety activities in mental health nursing. The results revealed a significant positive association between safety climate and patient safety activities, indicating that a positive safety climate within mental health settings is conducive to the implementation of activities aimed at promoting patient safety.

Furthermore, our study identified safety control as a key mediator in this association. Safety control refers to the perception of individuals regarding their ability to influence safety-related outcomes through their actions and behaviors. These results indicate that mental health nurses who perceive greater control over safety processes are more likely to engage in patient safety activities, despite a positive safety climate.

These results have important implications for healthcare organizations and policymakers seeking patient safety improvement in mental health settings. Healthcare organizations promote a culture of safety that encourages proactive engagement in patient safety activities by fostering a positive safety climate and providing mental health nurses with opportunities to exert control over safety-related processes.

This study revealed that gender, age, marital status, educational background, psychiatric experience, subjective health status, and job satisfaction did not affect patient safety activities. However, it was not consistent with the results of previous studies on mental nurses that the total work experience [5,34] affected patient safety management activities. This does not indicate that psychiatric work experience does not affect patient safety management activities, but that the psychiatric work experience of the study participants was relatively high at 75.1% for more than 3 years, 24.3% for more than 10 years, and 75.1% for those over 31 years of age; thus, age or psychiatric experience would not have affected patient safety activities. Additionally, in the patient safety activities of the study participants, the average of 3.90 and 3.95 points out of 5 were high, confirming many skills.

Safety awareness was the crucial factor in patient safety management for mental nurses through the above results. Thus, patient safety and the use of standardized regulations and mental guidelines to conduct nursing interventions need to be monitored to prevent safety accidents and to improve the patient safety activities of mental hospital nurses.

Additionally, a shortage of staff is a major problem in the field of mental health nursing. It causes stress, anxiety, and depression for nurses; in turn, these effects affect patient safety negatively [29,35]. Having an adequate number of staff and resources used for handling workload and work hours reportedly improves patient safety by approximately 2–3 times [36]. Therefore, sufficient manpower is essential to ensure patient safety and patient safety climate and safety control must be established.

## 5. Limitations

The study “The Safety Climate and Patient Safety Activities in Mental Health Nurses: The Mediating Effect of Safety Control” investigates the relationship between safety climate, patient safety activities, and safety control among mental health nurses. Here are some potential limitations of this study: First, the study has a limited sample size or has been conducted in a specific geographical area or healthcare setting, which could affect the generalizability of the findings to broader populations of mental health nurses. Second, the study has relied on self-reported measures to assess safety climate, safety control, and patient safety activities. Self-report measures can be subject to bias, such as social desirability bias or recall bias, which may affect the accuracy of the results. Third, the findings of the study may be specific to the context in which it was conducted, such as the organizational culture, policies, and resources available in healthcare.

## 6. Conclusions

Based on the study results, the following suggestions are made. First, nurses working at mental hospitals in some areas were selected through convenient sampling. Therefore, it is important to generalize the study results. Replication research on mental nurses working in various areas needs to be conducted. Second, hospitals need to support the establishment of a positive safety climate in terms of policy to promote mental health nurses’ patient safety nursing activities. Third, a nursing intervention program needs to be developed to improve mental health nurses’ safety control and verify the effects of the program.

These results emphasized the importance of organizational safety control measures and the creation of a positive safety atmosphere within mental healthcare settings. Such measures not only promote patient safety but also encourage mental health nurses to actively participate in activities that enhance patient well-being. Further research may explore additional factors that contribute to the remaining variance in patient safety nursing activities, thereby ultimately contributing to a more comprehensive understanding of patient safety in mental healthcare environments.

In conclusion, our study emphasizes the importance of safety climate and safety control in promoting patient safety activities among mental health nurses [7,29]. By understanding the mechanisms underlying these relationships, healthcare organizations can effectively mitigate risks. These findings have implications for healthcare organizations seeking to enhance patient safety and engage their nursing workforce in proactive patient safety initiatives.

## Figures and Tables

**Table 1:** Patient Safety Activities Based on General Characteristics (n = 177).

Characteristics	Category	n	%	Patient Safety Activities	
				Mean ± SD	t/F (*p*) Scheffé Test
Gender	Male	21	11.9	3.90 ± 0.58	0.30 (0.76)
	Female	156	88.1	3.86 ± 0.04	
Age	≤30	44	24.9	3.82 ± 0.47	1.39 (0.78)
	31–40	65	36.7	3.85 ± 0.45	
	41–50	46	26	3.90 ± 0.46	
	≥51	22	12.4	3.93 ± 0.60	
Marital Status	Single	73	41.2	3.83 ± 0.49	−0.99 (0.32)
	Married	104	58.8	3.90 ± 0.46	
Education	College	92	52	3.92 ± 0.48	1.58 (0.11)
	University	85	48	3.81 ± 0.47	
Carrier of Psychiatric Ward (Year)	<3	44	24.9	3.83 ± 0.51	0.39 (0.76)
	3–6	50	28.2	3.83 ± 0.38	
	6–10	40	22.6	3.90 ± 0.05	
	≥10	43	24.3	3.91 ± 0.52	
Subjective Health status	Good	87	49.2	3.87 ± 0.49	0.25 (0.77)
	Moderate or Poor	90	50.8	3.87 ± 0.04	
Violence Experience within 1 month	Have	99	55.9	3.91 ± 0.49	1.48 (0.14)
	Have not	78	44.1	3.81 ± 0.45	
Job Satisfaction	Satisfaction	95	53.7	3.89 ± 0.46	0.29 (0.74)
	Moderate or Dissatisfaction	82	46.3	3.83 ± 0.48	

**Table 2:** Degrees of Nurses’ Safety Climate, Safety Control, and Patient Safety Activities (n = 177).

Variable	Mean ± SD	Actual Range	Reference Range
Safety Climate	3.48 ± 0.54	2.18~4.78	1–5
Hospital contribution	3.37 ± 0.63	1.91~4.83	
Work-unit contribution	3.54 ± 0.57	2.19~4.90	
Safety Control	3.65 ± 0.80	1.87~5.00	1–5
Patient Safety Activities	3.87 ± 0.47	2.81~5.00	1–5
Patient identification	3.83 ± 0.72	2.16~5.00	
Prescription and medication	3.87 ± 0.61	2.50~5.00	
Environment safety	3.73 ± 0.83	1.66~5.00	
Infections, falls, and pressure ulcers prevention	3.88 ± 0.60	2.42~5.00	
Coping emergency	3.90 ± 0.64	2.25~5.00	
Coping with psychiatric problems and situation	3.95 ± 0.73	2.00~5.00	

**Table 3:** Correlations between Safety climate, Safety Control, and Patient Safety Activities (n = 177).

Variables	Safety Climate r (*p*)	Safety Control r (*p*)	Patient Safety Activities r (*p*)
Safety climate	1		
Safety control	0.40 (<0.001)	1	
Patient safety activities	0.40 (<0.001)	0.43 (<0.001)	1

**Table 4:** Mediating effect of safety control, on the relationship between the study participants’ safety climate and patient safety activities (n = 177).

Step	Independent Variables	Dependent Variables	B	S.E	β	t (*p*)	R^2^	Adjusted R^2^	F (*p*)
1	Safety climate	safety control	0.63	0.10	0.42	6.29 (<0.001)	0.184	0.180	39.56 (<0.001)
2	Safety climate	patient safety activities	0.34	0.06	0.39	5.71 (<0.001)	0.157	0.152	32.66 (<0.001)
3	Safety climate Safety control	patient safety activities	0.22	0.06	0.26	3.56 (<0.001)	0.238	0.229	27.19 (<0.001)
			0.18	0.04	0.31	4.29 (<0.001)			

Sobel test: Z = 3.66, *p* < 0.001.

## Data Availability

The data presented in this study are available on request from the corresponding author.
